# Phenotypic diversity drives paracrine drug tolerance

**DOI:** 10.15252/emmm.201707956

**Published:** 2017-07-10

**Authors:** Thomas Kuilman, Daniel S Peeper

**Affiliations:** ^1^ Department of Molecular Oncology and Immunology The Netherlands Cancer Institute Amsterdam The Netherlands

**Keywords:** Cancer, Skin

## Abstract

Considerable clinical successes have been achieved in cancer treatment since the introduction of targeted therapies. However, almost inevitably tumors develop therapy resistance, which limits durable clinical responses. Tumors are often heterogeneous, and as a result, therapy‐resistant cells are present even before the start of treatment. Resistance is commonly mediated via genetic changes. However, in this issue of *EMBO Molecular Medicine*, Smith *et al* (2017) report that phenotypic heterogeneity can contribute to resistance as well.

The BRAF inhibitors developed for the treatment of BRAF mutant melanoma are among the posterchilds for targeted therapy. Whereas the majority of patients initially respond well to vemurafenib, or dabrafenib, most (albeit not all) eventually succumb to cancer relapse (Wong & Ribas, 2016). Although progression‐free survival increases upon co‐administration of a MEK inhibitor, even then the majority of patients fail to experience durable clinical benefit (Robert *et al*, 2015). Various genetic alterations have been described that underlie resistance to these targeted inhibitors, and they commonly result in reactivation of the MAPK pathway (Welsh *et al*, 2016). One of the difficulties in preventing or combating resistance is that even within individual patients, multiple resistance mechanisms are operational owing to intra‐ and inter‐tumor heterogeneity (Kemper *et al*, 2015).

Next to genomic changes, specific phenotypic states have also been correlated with both acquired and adaptive resistance (Konieczkowski *et al*, 2014; Müller *et al*, 2014). In particular, melanoma cells display either of at least two cellular states, termed “proliferative” and “invasive” (Hoek *et al*, 2006). They are characterized by high and low MITF/AXL ratios, and drug sensitivity and resistance, respectively. Recent single‐cell sequencing analysis confirms this pattern in patients (Tirosh *et al*, 2016). While melanoma cell lines tend to display either of the two phenotypes, there is evidence that melanomas *in vivo*, too, display phenotypic heterogeneity and plasticity (Hoek *et al*, 2008). In an elegant study in this issue of *EMBO Molecular Medicine*, Smith *et al* (2017) report that melanoma heterogeneity with respect to its phenotypic state plays an important role in treatment resistance.

The investigators examined the heterogeneity of the melanoma phenotypes upon treatment with a BRAF inhibitor, using MITF as readout. In fact, they and others had found previously that MITF expression in general increases upon treatment (Haq *et al*, 2013; Smith *et al*, 2016). Now, Smith *et al* (2017) report that melanoma cells display heterogeneous staining at the single‐cell level without enriching for an AXL‐high/MITF‐low population. Since cells that express basal MITF levels are known to be more responsive to BRAF inhibition, they hypothesized that MITF‐high cells may support the survival of those expressing lower MITF levels. Indeed, they found in a zebrafish xenograft model that BRAF inhibitor‐pretreated melanoma cells, expressing elevated MITF levels, are not only more resistant to BRAF inhibition themselves, but also decrease the sensitivity to BRAF inhibition of untreated cells. Thus, it appears that upon MAPK inhibitor treatment in a heterogeneous *in vivo* setting, treatment‐induced MITF‐high cells support the proliferation and survival of sensitive cells expressing lower MITF levels.

Investigating the mechanism for this phenomenon, the team discovered that the protective effect partially occurs in a paracrine manner. Moreover, this correlates with a restoration of ERK activity in co‐cultured cells. Using a proteomics‐based approach, the investigators show that many of the secreted factors that are specifically expressed by pretreated cells and that have the potential to activate the MAPK pathway are regulated by MITF. Upon depletion of MITF in the pretreated cells, the protective effect on non‐pretreated melanoma toward BRAF inhibitors was abrogated. Thus, heterogeneity in MITF expression not only characterizes distinct melanoma phenotypes, but also allows for the creation of a mini‐community in which cells with different properties influence one another's drug response profiles via paracrine signals.

By scrutinizing the subset of secreted factors that are targets of MITF, Smith *et al* (2017) found that via increased expression of endothelin‐converting enzyme‐1 (ECE1), which produces biologically active endothelin‐1 (EDN1), EDN1 is strongly induced in cells pretreated with BRAF inhibitor. Moreover, it allows for endothelin receptor B (EDNRB)‐dependent proliferation of cells of the proliferative phenotype in the face of BRAF inhibitor treatment. Expression of the related endothelin receptor A (EDNRA) receptor, which is predominantly expressed in AXL‐high/MITF‐low cells, surprisingly also contributes to antagonizing BRAF inhibition in cells with an invasive phenotype. Therefore, endothelin‐dependent signaling contributes to paracrine protection to MAPK inhibition.

Finally, Smith *et al* (2017) investigated whether this finding can be therapeutically exploited. First, using the pan‐EDNR inhibitor Bosentan, they show that combination treatment with BRAF inhibitor is more effective in interfering with the growth of melanoma xenografts than either single treatment. Importantly, they also see that AXL expression is lower in tumors that are treated with the combination compared to the BRAF inhibitor single‐agent treatment. Similarly, with specific inhibitors for EDNRA and EDNRB, inhibition of either endothelin receptor synergizes with BRAF inhibition, while these combination therapies suppress enrichment of the dangerous AXL‐high cells.

By focusing on the biology of melanoma cells in the context of standard‐of‐care targeted treatment, Smith *et al* (2017) draw a number of intriguing conclusions. First, they show that specific sub‐populations within a heterogeneous tumor have the potential to support the survival of others (Fig 1). While similar observations have been made for “cooperative” tumor invasion (Chapman *et al*, 2014), the concept that therapy resistance is fueled by signaling between phenotypically different populations has interesting ramifications. For instance, it is possible that treatment with BRAF inhibitors actively selects for heterogeneous tumors, where the different sub‐populations support each other's survival. Indeed, heterogeneous MITF staining in patients on treatment is commonly observed, which could point toward selective pressure driving heterogeneity.

**Figure 1:**
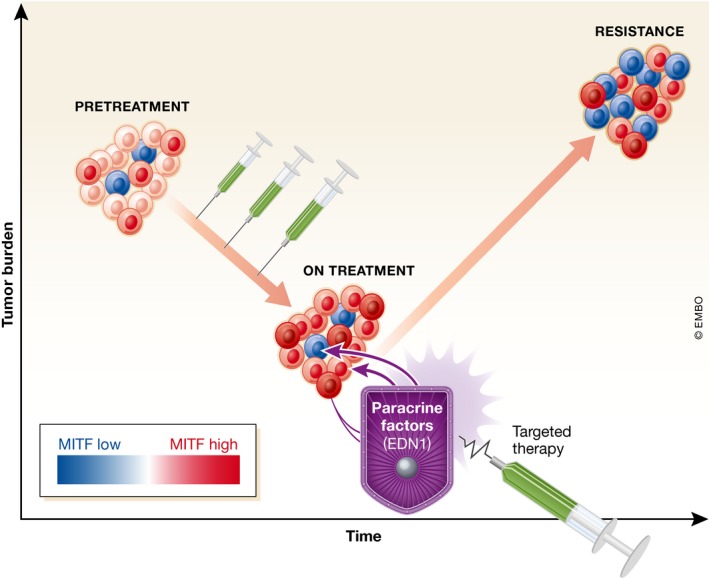
Phenotypic diversity drives paracrine drug tolerance Melanoma responds to BRAF inhibitor treatment by increasing bulk‐MITF expression, while maintaining heterogeneity. MITF‐high cells (dark red) in this context express endothelin‐1, which promotes the survival and proliferation of cells expressing basal (pink) or low (blue) levels of MITF. This contributes to the emergence of resistant (MITF‐low) tumors.

From a conceptual point of view, the paracrine protective mechanism is an interesting trait that raises a number of new questions. For instance, do MITF‐low cells also support MITF‐proficient populations via a similar mechanism? Is endothelin‐mediated tumor cell support just the tip of the iceberg and are many more factors contributing to paracrine support? Finally, these results beg the question whether in the absence of treatment similar mechanisms contribute to tumorigenesis. Speaking in favor of such a mechanism are data from lung cancer, for which tumor heterogeneity has been shown to create a symbiotic microenvironment in which paracrine signaling between subclones drives a particular phenotype, in this case metastasis (Kwon *et al*, 2015).

The findings are also of potential interest from a clinical perspective. While melanoma cells of the proliferative phenotype display a broad treatment sensitivity profile, it is the AXL‐high, invasive, and metastatic cells that are intrinsically difficult to target. Since Smith *et al* (2017) show that the support mechanism is mediated by paracrine factors, and because these are relatively easily pharmacologically tractable, therapeutic intervention to prevent early BRAF inhibitor resistance might become a realistic option. Combined EDNR/BRAF inhibition targets both MITF‐proficient and MITF‐deficient cells, and there is apparent pressure against AXL‐high cells relative to BRAF inhibitor monotherapy. While clinical implementation of these findings will require further in‐depth studies on this topic, the report by Smith *et al* (2017) is a significant step forward in our understanding of the biology of melanoma on BRAF inhibitor treatment and also suggests that novel combination therapies may be designed at preventing early resistance.
